# Bimetallic layered-double hydroxides anchored on reduced graphene oxide as a bifunctional electrocatalyst for electrochemical water splitting

**DOI:** 10.1039/d5ra04536c

**Published:** 2025-09-18

**Authors:** Asad Ullah Khan, Syed Haider Ali Shah, Fariah Salam, Afzal Shah, Faiza Jan Iftikhar, Muhammad Umar Farooq, Muhammad Abdullah Khan

**Affiliations:** a Department of Chemistry, Quaid-i-Azam University Islamabad 45320 Pakistan afzals_qau@yahoo.com; b Renewable Energy Advancement Laboratory, Department of Environmental Sciences, Quaid-i-Azam University Islamabad 45320 Pakistan; c NUTECH School of Applied Science & Humanities, National University of Technology Islamabad 44000 Pakistan; d National Center for Physics Islamabad 45320 Pakistan

## Abstract

Developing efficient electrocatalysts for both the hydrogen evolution reaction (HER) and the oxygen evolution reaction (OER) is essential for advancing a sustainable energy future. In this study, bimetallic NiMo–LDH nanoflakes were successfully integrated onto reduced graphene oxide using a straightforward hydrothermal method. The composite material was characterized by FTIR, XRD, SEM, EDS, and XPS. Electrochemical evaluations highlighted the impact of rGO content, revealing that the NiMo–LDH with 7% rGO exhibited superior performance in OER, requiring only 230 mV overpotential and a Tafel slope of 60 mV dec^−1^ at a current density of 10 mA cm^−2^ in 1.0 M KOH. This enhanced performance is attributed to improved charge separation and transfer at the electrocatalyst/electrolyte interfaces, driven by the synergistic effects of rGO and NiMo–LDH. Furthermore, the composite electrocatalyst showcased bifunctional capabilities, with promising HER performance characterized by favorable overpotential and Tafel slope in 0.5 M H_2_SO_4_. Long-term stability was confirmed through chronoamperometry, while electrochemical impedance spectroscopy revealed efficient charge transport across the modified glassy carbon electrode represented as NiMo–LDH@rGO/GCE. Additionally, the synthesized catalyst demonstrated good recoverability, exhibiting appealing onset and overpotentials.

## Abstract 2

Developing efficient water splitting electrocatalysts is essential for advancing a sustainable energy future.

## Graphical abstract

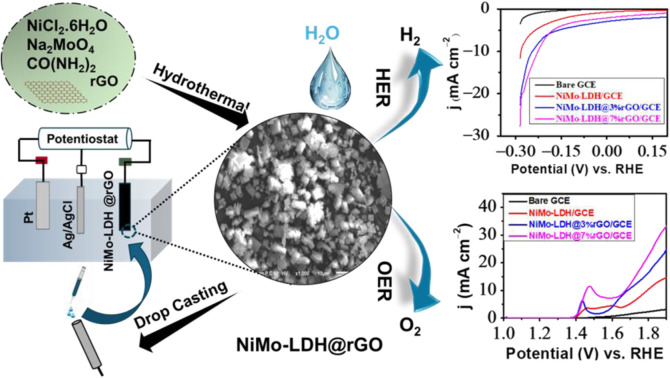

## 1. Introduction

Increasing global energy demand and the resulting environmental issues make the pursuit of renewable and clean energy sources imperative as viable alternatives to fossil fuels. However, sustainable energy sources, such as solar and wind, are intermittent and unpredictable, depending on natural weather conditions.^1^ Hence, to ensure a reliable supply, it is pertinent to effectively store energy as chemical energy. Green hydrogen is widely considered an energy vector for future sustainable energy systems owing to its significant energy density, zero carbon emissions, and recyclability. It is predominantly generated through steam reformation reactions under high temperature conditions, resulting in harmful CO_2_ emissions.^2^ This process is energy-intensive and leads to global warming, hence, alternatives that involve green hydrogen production *via* electrolysis are being pursued. Electrocatalytic water splitting generates high-purity hydrogen by using electricity under mild conditions, producing water as an innocuous byproduct. It is a promising and scalable method that enhances the overall energy efficiency of hydrogen generation. However, it is more expensive than thermo-catalytic processes on account of its low conversion efficiency, limited scalability, and input of expensive materials.^3^

In principle, electrocatalytic water splitting comprises two-half reactions, the HER and OER. OER exhibits more sluggish kinetics compared to HER due to its four-electron-proton transfer process. Therefore, it is considered as the bottleneck in water splitting processes. To facilitate the kinetics of these reactions, a strategic design of electrocatalysts is crucial for enhancing reaction kinetics, particularly for OER.^4^ Noble metal-based catalysts such as Pt exhibit exemplary HER performance characterized by low overpotentials and small Tafel slopes, while Ru and Ir-based catalysts are used for OER due to their exceptional durability.^5,6^ The main issues associated with these electrocatalysts stem from their high cost and scarcity in the Earth's crust, which significantly hamper their extensive applications in strongly alkaline and acidic environments found in electrolyzers and proton exchange membranes (PEMs), respectively. Consequently, persistent endeavors have been directed toward development of economical bifunctional electrocatalysts that are efficient for both HER and OER.^7^ These electrocatalysts include transition metal (TM)-based oxides, hydroxides, sulphides, phosphides, oxyhydroxides, and selenides. Among the transition-based catalysts, 2D layered-double hydroxides (LDHs) have emerged as significant alternatives for OER due to the presence of OH^−^ in the host layers.^8^ The structure of LDH provides active centers for OER to take place, where electrons occupy the d-orbitals of the TMs, thus enhancing the redox properties during the water oxidation process. The exposure of these sites in LDHs promotes coordination between the d-orbitals of the TM cations and oxygen species that play a vital role in lowering the energy barriers and speeding up the electrochemical reaction and ion-trapping ability. The anions between the layers maintain the equilibrium charge on surface hydroxides while at the same time preserving structural integrity.^9^ As a result, 2D LDH nanomaterials have emerged as prominent electrocatalysts in recent years owing to their distinctive layered architecture and adaptable properties. Further, the TM ions in LDH materials can provide vacant orbitals or lone electron pairs that influences the electron migration rate and the reconfiguration of the electrocatalyst surface.^10^ For example, Ni species offer a substantial number of occupied orbitals containing lone pair electrons, whereas high-valent molybdenum (Mo^3+/^Mo^4+^) seems to be an exceptional candidate for supplying empty orbitals with minimal or no occupation of d orbitals, asserting it as crucial for catalytic and ligand bonding reactions. Consequently, Ni and Mo-based nanomaterials can enhance active sites and inherent catalytic activity. Moreover, modifying the LDH intercalation anions can significantly mitigate the poisoning of the electrocatalyst surface.^11^ Ma and his coworkers successfully adopted a co-doping strategy by using Ru and Mn bimetallics to facilitate the systematic construction of electronic structure of both NiCo–LDH and NiCoP.^12^ Typically, LDH nanomaterials tend to agglomerate, with the active sites situated at the edges, which influences the interaction between the active sites and electrolyte leading to the constrained activity of LDHs for overall water splitting. However, the electrical conductance, functionality, surface area, and stability of TM hydroxides are significantly enhanced by introducing carbon-based materials due to their synergistic effects.

Carbon materials, such as graphene and other nanocarbon forms, have gained interest due to their surface functionality and electrocatalytic applications. This is largely attributed to their strong π–π interactions, which facilitate rapid electron mobility and enhance stability.^13,14^ Specifically, graphene oxide (GO) is an economical carbon-based conductor that improves catalytic characteristics. The additional reduction of GO to reduced graphene oxide (rGO) leads to improved electrical conductivity and significantly increases the surface area of metal hydroxides, facilitating enhancement in current and enabling rapid charge transfer. Moreover, the π-network in rGO contributes to the characteristic properties of graphene. Further, there has been a growing trend in the development of bifunctional electrocatalysts.^15^ A notable example is the rational design of a 2D heterostructure composed of graphene and hexagonal boron nitride (h-BN), which facilitates the formation of abundant interfaces and electroactive sites through π–π interactions. This catalyst demonstrated impressive performance, achieving a low overpotential of 28 mV at 10 mA cm^−2^ for the HER and 360 mV at 50 mA cm^−2^ for the OER. Additionally, it effectively served as both the cathode and anode in a quasi-triangulated bipolar electrolysis system, efficiently promoting overall water splitting.^16^ In addition, Rashid *et al.*, employed an environmentally safe, stable, and cost-effective NiCo nanocomposite functionalized over rGO electrocatalyst to achieve efficient HER and OER activities at low overpotentials, resulting in a benchmark density of 10 mA cm^−2^.^17^ Sim and his colleagues synthesized a heterostructure composed of covalently bound 3D MoSe_2_ and rGO, which resulted in increased number of edge sites for improved catalytic properties for both OER and HER.^18^ Moreover, Sannegowda and his research team synthesized iron phthalocyanine by incorporating 4-amino-3-napthalene-2-sulfonic acid tri-functional monomers, and characterized it by employing a range of spectroscopic and analytical techniques. The prepared supramolecule was integrated with rGO, which was then immobilized onto a GCE for assessment of its bifunctional activity for both HER and OER during water electrolysis.^19^ Similarly, Gaddaerappa and his research group reported a highly promising bifunctional electrocatalyst synthesized by using quinone substituted cobalt(ii) phthalocyanine (HQCoPc) in conjunction with carbon nanoparticles, which demonstrated high electrocatalytic performance for HER as well as OER.^20^

This study aims to synthesize a bimetallic NiMo–LDH nanomaterials, which is a hollow configuration superstructure synthesized by employing a hydrothermal method to accelerate water splitting reactions. With careful tuning of Ni and Mo content, the resulting bimetallic NiMo–LDH exhibited notable catalytic performance for water splitting. To make bimetallic LDH most efficient, stable and bifunctional, it was supported on rGO to form a hybrid nanomaterial. Experimental investigations indicated that the nanostructured heterostructure rGO-anchored, NiMoO_4_/NiFe–LDH nanosheet catalyst exhibited favorable kinetics and rapid charge and mass transfer while providing active sites with optimized adsorption energy and a tailored electronic structure while heteroatom doping with Mo in NiFe–LDH and heterostructuring with NiS_*x*_ boosts its bifunctional activity for OER and HER.^21,22^ Consequently, it is expected that bimetallic Mo–NiFe LDH nanomaterial can demonstrate significantly enhanced HER and OER performance. Furthermore, extensive characterization techniques were utilized to examine physical and chemical properties. Further, the morphology, electronic structure, catalytic performance, and mechanism of the rGO-anchored bimetallic electrocatalysts were investigated by varying the quantity of rGO (3% and 7%). This novel material exhibits high efficiency for hydrogen production-a greener fuel that can contribute toward a carbon-free hydrogen economy. Furthermore, to the best of our knowledge, 3% and 7% NiMo–LDH@rGO hybrid nanomaterial have not been utilized as an electrocatalyst for water splitting before, nor have they been recorded for any other applications in scientific literature.

## 2. Materials and methods

### 2.1 Chemicals

The chemicals employed for synthesis include NiCl_2_·6H_2_O, H_2_SO_4_, urea, sodium molybdate (Na_2_MoO_4_), ethanol, NaNO_3_, KOH, and graphitic natural flakes (GNF). Deionized water was used in all experiments. Analytical grade chemicals were procured from Sigma-Aldrich and used without further purification.

### 2.2 Characterization

XRD was conducted within a range of 0° to 80° by employing a PANalytical diffractometer model 3040/60 X′ Pert PRO running at 45 kV and 40 mA with Copper's kα radiation (*λ* = 0.154 nm) as an X-ray radiation source, with a step size of 0.025°. Similarly, the Nicolet Summit FTIR spectrometer was employed for compositional analysis of the materials. Moreover, the surface morphology and elemental composition were evaluated using the “ZEISS” at an operating VOLTAGE of 15 kV using a field emission scanning electron microscope (FESEM) and Energy Dispersive X-ray Detector (EDX). Omicron was used to analyze the electronic state of the materials by X-ray photoelectron spectroscopy (XPS). Electrochemical assessments were conducted by using a Gamry workstation (IFC 5000E Potentiostat/Galvanostat/ZRA) in an electrochemical cell containing 1.0 M KOH (pH = 13.9), with an immersed modified GCE as a working electrode, platinum as a counter electrode and Ag/AgCl as a reference electrode.

### 2.3 Synthesis of NiMo–LDH and NiMo–LDHs@rGO

Hydrothermal method was employed for the synthesis of NiMo–LDH nanosheets while rGO was synthesized using the modified Hummer's method. 1.1 mmol of NiCl_2_·6H_2_O, 0.5 mmol of Na_2_MoO_4_, and 6 mmol of CO(NH_2_)_2_ were individually dissolved in 100 mL deionized water and vigorously mixed to obtain a homogeneous solution. The solution was subsequently transferred to an autoclave lined with Teflon. Following sealing, it was placed in an electric oven and heated at 120 °C for 4 hours. Samples were carefully rinsed using deionized water and ethanol and allowed to cool at room temperature. The resultant product collected *via* vacuum drying at 60 °C overnight, was labeled as NiMo–LDH.^23^

**Fig. 1:**
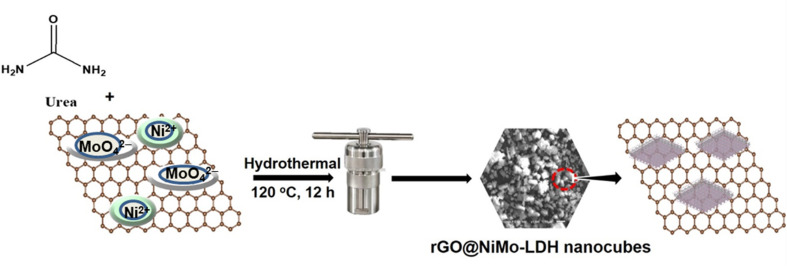
Systematic depiction of the synthesis of NiMo–LDH@rGO.

Similarly, in a typical method, 2 mmol of NiCl_2_·6H_2_O, and 1 mmol of Na_2_MoO_4_·2H_2_O were mixed in 50 mL of distilled water as shown in Fig. 1. The mixture was then treated with *x* wt% rGO (where *x* = 3 and 7) along with 12 mmol of urea, and continuously stirred. Subsequently, the solution was placed in an autoclave and subjected to a temperature of 120 °C for 12 hours, leading to the formation of a black precipitate. Upon cooling to room temperature, the synthesized samples were washed with a water/ethanol mixture and dried overnight at 60 °C in a vacuum drying oven. Finally, NiMo–LDH@rGO nanoflakes were obtained. Additionally, pristine NiMo–LDH was synthesized using the hydrothermal method, without adding the rGO, as a control.

## 3. Electrode modification and acquisition of electrochemical data

A three-electrode configuration was employed, consisting of a glassy carbon working electrode, an Ag/AgCl (sat. KCl) reference electrode, and a platinum wire counter electrode. All electrochemical assessments were conducted using a Potentiostat/Galvanostat/ZRA 02529 (Interface 5000E) from Gamry, located in Warminster, PA, USA. The electrocatalytic performance of the NiMo–LDH@rGO catalysts for the oxygen and hydrogen evolution reactions was analyzed in 1 M KOH (pH 13.9) and 0.5 M H_2_SO_4_ (pH 0.3), respectively. Initially, the GCE substrate (with 5 mm diameter) was polished with alumina powder and a rubbing pad. Thereafter, it was rinsed with deionized water, and dried in an oven. The LDH was formulated as an ink by combining 3.5 mg of LDH with 250 μL of DMF. Following 30 minutes of sonication, homogeneous ink was made and applied to the GCE *via* drop casting, with an LDH catalyst loading maintained at 0.4 mg cm^−2^. Linear sweep voltammetry (LSV) was utilized as the electrochemical technique using a scan rate of 10 mV s^−1^. Potentials were calibrated to the relative hydrogen electrode scale (*E*_RHE_ = *E*_Ag/AgCl_ + 0.197 + 0.059 × pH) and adjusted for IR drop. ElS data was acquired over a frequency range from 0.1 Hz to 100 kHz utilizing fixed potentials of 1.5 V and −0.41 V for the OER as well as HER. The collected data were analyzed using a Randles equivalent circuit, and the charge transfer resistance was determined. In the equivalent circuit, *R*_s_ represents the solution resistance of the electrolyte, electrocatalysts, and all the contacts, while *R*_ct_ denotes the charge transfer resistance between the catalysts and electrolyte. The smaller *R*_ct_ value is associated with efficient electron transfer. The stability of the chosen bimetallic LDH supported on rGO (NiMo–LDH@rGO) was investigated by running LSVs before and after 24 hours of chronoamperometric operation at a constant potential for a current density of 10 mA cm^−2^.

The eqn (1) and (2) were employed to determine the overpotential (*η*) for OER and HER.

1 *η*_OER_ = *E*_RHE_ − 1.23

2 *η*_HER_ = 0 − *E*_RHE_

### 3.1 Tafel slope

The Tafel slope was obtained by analyzing the kinetics of the reactions. The subsequent eqn (3) was employed to produce a Tafel plot by making use of LSV data.

3 *η* = *a* + *b* log *j*

In the above equation, *a* and *b* demonstrate the charge transfer coefficient and Tafel slope, and *j* and *η* are the current density and overpotential, respectively.

### 3.2 Electrochemical active surface area (ECSA)

In evaluating the electrochemical activity, it is essential to take into account the ECSA through cyclic voltammograms (CV) in the non-faradic region. This was to investigate the polarization behavior in the non-faradic region in a voltage range (1.0–1.17 V) at various sweep rates, ranging from 10 to 50 mV s^−1^. Subsequently, a relationship between Δ*j* and sweep rate was determined by the slope of the graph obtained *via* a linear fit.^24^ Moreover, the double layer capacitance (*C*_dl_) value was determined, which is equivalent to twice the computed slope of the graph, as indicated in eqn (4). Further, by using eqn (5), ECSA was determined for the flat electrode by dividing *C*_dl_ with specific capacitance (*C*_sp_). Previous research indicates that the *C*_sp_ value of a flat electrode generally ranges from 0.01–0.05 mF cm^−2^, with a value of 0.04 mF cm^−2^ being selected for the current analysis.4
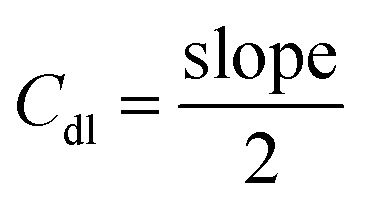
5
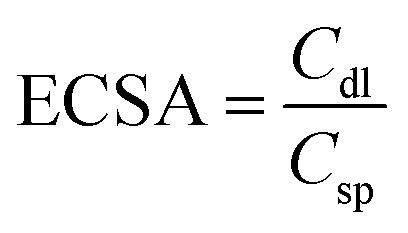


The normalization of the curve was performed for the ECSA to find the true efficiency of the prepared electrode as porosity and roughness of the surface affect the catalytic efficiency. Thus, the electrochemical surface area, was calculated using the prescribed formula given in eqn (6) by using normalized current density (*j*_s_) for determining the catalytic activity of the electrodes.6
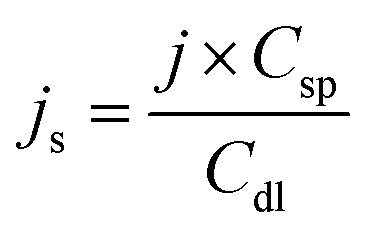


### 3.3 Mass activity

This term is commonly employed to characterize the catalytic efficiency related to the loaded mass and the geometric area of the active electrode. The more mass activity, the greater the catalyst performance. The catalyst's mass activity for water splitting is influenced by its composition, crystal structure, surface area, and electrical properties and is calculated by using eqn (7).7
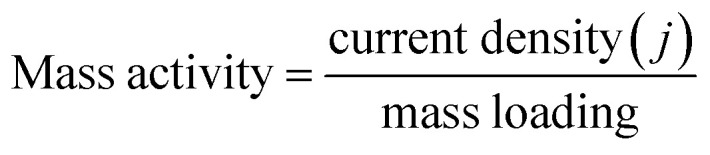


### 3.4 Turnover frequency (TOF)

Catalysts play a vital role in accelerating the electron transfer processes. This ability is represented by a kinetic parameter known as TOF that reflects the efficiency and performance of an electrocatalyst. Determining the TOF values for HER and OER is widely used to determine the efficiency of catalysts and thus represents the rate at which the catalysts can facilitate the electrochemical reaction.^22,25^ The turnover frequency values for both the half reactions are calculated using eqn (8) and (9).8
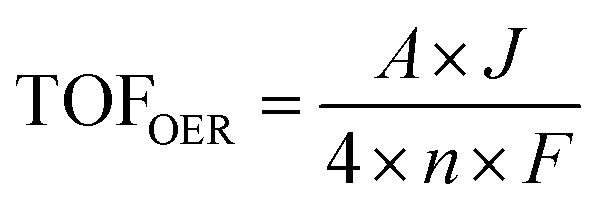
9
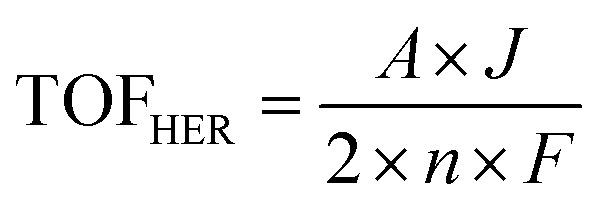


## 4. Results and discussion

**Fig. 2:**
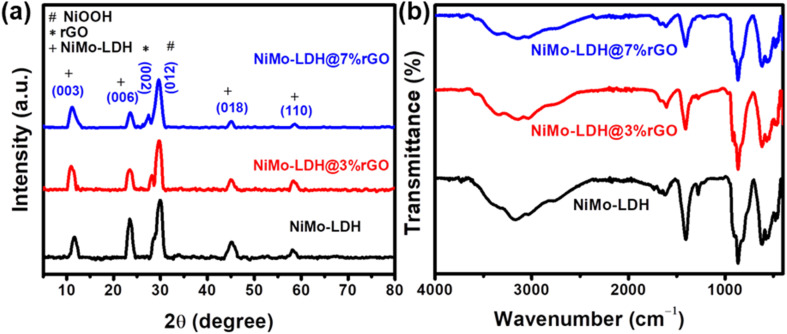
(a) XRD patterns of NiMo–LDH, NiMo–LDH@3%rGO, and NiMo–LDH@7%rGO. (b) FTIR spectra of NiMo–LDH, NiMo–LDH@3%rGO, and NiMo–LDH@7%rGO.

XRD was employed for phase purity and structural analysis. Fig. 2(a) shows XRD pattern of NiMo–LDH, NiMo–LDH@3%rGO, and NiMo–LDH@7%rGO. The corresponding peaks at 11.7°, 23.5°, 30°, 45°, and 58.4° confirmed the presence of elements constituting NiMo–LDH and are attributed to (003), (006), (012), (018), and (110) Miller planes. Similarly, the peak at 27.1° corresponds to (002) plane, confirming the presence of rGO in the as-prepared electrocatalysts. These peaks exactly match with JCPS card no. 01-082-8040. Moreover, integration of rGO results in enhancing amorphosity of the materials, which is also clear from XRD micrographs by observing a reduction in sharpness of peaks and increase in peak broadening. Similarly, FTIR as shown in Fig. 2(b) was employed for compositional analysis, and to verify the functional groups present in rGO-supported NiMo–LDH. In the NiMo–LDH@rGO spectrum, the absorption band around 3000 cm^−1^ to 3300 cm^−1^, corresponds to OH stretching vibration.^26^ Similarly, the adsorption band around 1600 cm^−1^ and 1407 cm^−1^ corresponds to the bending mode of water molecules and CO^3−^ stretching vibration, respectively.^11^ The CO_2_^3−^ is intercalated in the LDH, and provides stability to the material.

**Fig. 3:**
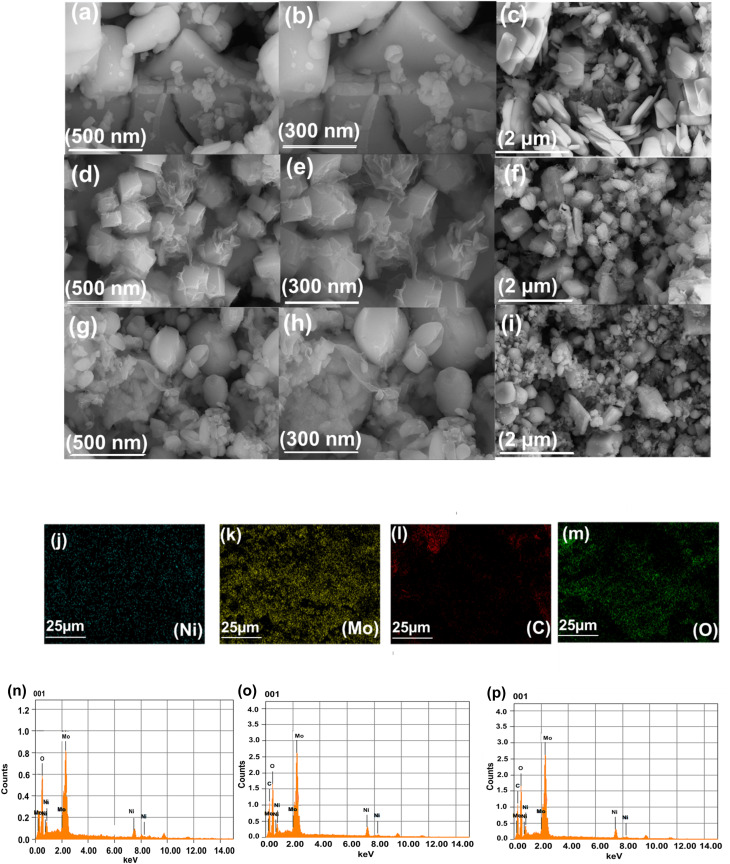
FE-SEM images of (a–c) NiMo–LDH at 500 nm, 300 nm, 2 μm (d–f) NiMo–LDH@3%rGO at magnifications of 500 nm, 300 nm, 2 μm (g–i) NiMo–LDH@7%rGO 500 nm, 300 nm 2 μm. (j–m) Elemental mapping of Ni, Mo, C, O. (n–p) EDX spectra of NiMo–LDH, NiMo–LDH@3%rGO and NiMo–LDH@7%rGO respectively.

FE-SEM is most commonly used for characterization of solid materials and analysis of surface morphology. It offers magnification ranging from 10 to 300 000 times and enables non-destructive analysis. With a spatial resolution of up to 500 nm, FE-SEM was employed to analyze the surface morphology of the synthesized materials at various resolutions. The FE-SEM images presented in Fig. 3 illustrate the morphological changes of NiMo–LDH as the rGO content varies from 3% to 7%. The pure NiMo–LDH in Fig. 3(a) exhibits a characteristic nanoflake-like aggregates. Upon addition of 3% rGO (Fig. 3(d)–(f)), the texture transitions to a more porous form, suggesting the integration of rGO and an increase in the spacing between LDH layers. At 7% rGO (Fig. 3(g)–(i)), a significantly more open and interconnected network is observed, indicating that the higher rGO content successfully prevents the restacking of LDH. Similarly, EDS analysis was performed, which confirmed the presence of Ni, Mo, C and O in the synthesized nanomaterials as show in Fig. 3(j)–(p).

**Fig. 4:**
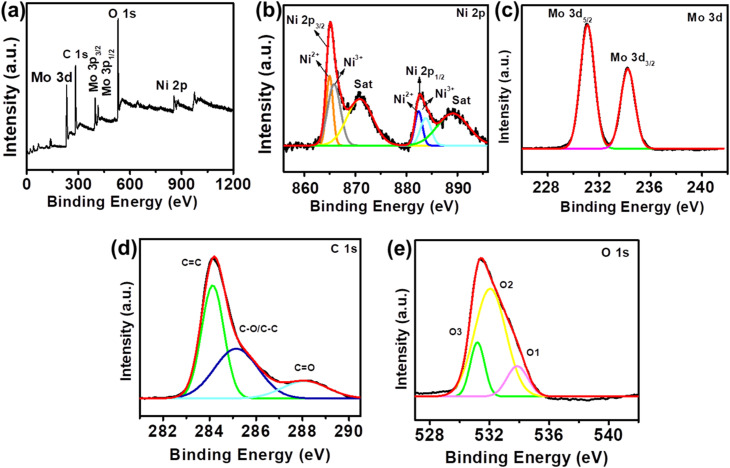
(a) XPS full survey spectrum of NiMo–LDH@rGO. High resolution spectra of (b) Ni 2p. (c) Mo 3d. (d) C 1s. (e) O 1s.

Similarly, the elemental composition and electronic state of the materials were examined by using XPS. The XPS spectrum clearly depicts the presence of various elements including Ni, Mo, C and O, which exactly aligns with the results obtained from EDS mapping as shows in Fig. 4(a). As depicted in Fig. 4(b) the Ni 2p spectrum of NiMo–LDH@rGO nanomaterial can be deconvoluted into four peaks, which consist of two main peaks due to spin–orbit doublets and two satellite peaks. The main peaks correspond to the Ni 2p_3/2_ and Ni 2p_1/2_ orbitals, respectively, which confirm the coexistence of Ni^3+^ and Ni^2+^. Moreover, the peak separation obtained between Ni 2p_3/2_ and Ni 2p_1/2_ is around 17.5 eV signifying their presence in the respective states.^27,28^ Likewise, in the Mo 3d Spectrum shown in Fig. 4(c) the two distinctive peaks were ascribed to the Mo 3d_5/2_ and Mo 3d_3/2_, respectively, which confirms the oxidation state of Mo^6+^. These states are found at binding energy levels of approximately 231.1 eV and 234.2 eV, respectively. The energy separation between these two peaks, amounts to 3.1 eV, highlighting the typical splitting between the spin states.^29^Fig. 4(d) depicts a high-resolution C 1s spectrum, in which various oxygen functional groups are clearly associated with carbon. The non-oxygenated ring link with carbon (C=C bond), has a peak at 284.1 eV; the epoxy and hydroxyl groups have a peak at 285.2 eV; and the O=C–OH linkage associated with the carboxyl group has a peak at 288.1 eV.^30^Fig. 4(e) shows the O 1s spectra of the NiMo–LDH@rGO nanocomposite, which is deconvoluted into three separate peaks at 531.2 eV (O–Ni/O–Mo), 532.1 eV (O–C), and 533.8 eV (O=C). The presence of these oxygen species implies the presence of metal oxygen and oxygen-containing groups in both the LDH and rGO components.^31^

### 4.1 Electrochemical water oxidation

**Fig. 5:**
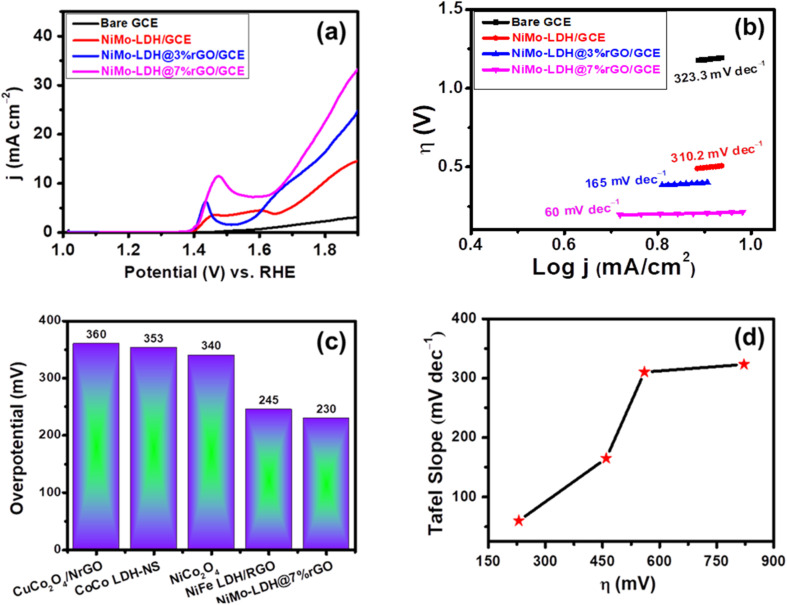
(a) LSV curves were obtained at bare GCE, NiMo–LDH/GCE, NiMo–LDH@3%rGO/GCE, and NiMo–LDH@7%rGO/GCE in 1.0 M KOH solution (b) Tafel plot was obtained at rGO-based LDH in 1.0 M KOH solution (c) illustration of overpotential for different electrocatalysts. (d) Relationship with overpotential and Tafel slope.

Following the successful synthesis and characterization of the nanomaterials, a series of investigations on their electrocatalytic activity were conducted at GCE. Fig. 5(a) displays linear voltammetric scanning curves of the three synthesized catalysts, NiMo–LDH/GCE, NiMo–LDH@3%rGO/GCE, and NiMo–LDH@7%rGO/GCE. The reference point for assessing the catalytic performance of the nanomaterials was established at a current density of 10 mA cm^−2^. At this specific current density, the overpotentials recorded were 540 mV for NiMo–LDH, 460 mV for NiMo–LDH@3%rGO, and 497 mV for NiMo–LDH@12%rGO (see Fig. 5(a) and S1). Nonetheless, the synthesized NiMo–LDH@7%rGO has overpotential of merely 230 mV at 10 mA cm^−2^, significantly lower than that of NiMo–LDH@3%rGO, NiMo–LDH@12%rGO and other reported electrocatalysts (see Table 1). Moreover, the bimetallic NiMo–LDH shows a peak which is attributed to a change in its oxidation state as depicted in Fig. 5(a) at 1.4 V *vs.* RHE. Here, Ni goes from lower Ni^2+^ oxidation state to Ni^3+^ showcasing high activity for OER^32^ likely attributed to the redox reaction of Ni^2+^/Ni^3+^ within the catalyst and also corroborates well with the XPS results.^33^

**Table 1:** Comparison of reported OER electrocatalysts with the as-synthesized material: NiMo/GCE, NiMo–LDH@3%rGO/GCE, and NiMo–LDH@7%rGO/GCE

Catalysts	Electrolyte	*η* (mV)	Tafel slope (mV dec^−1^)	Ref.
NiFe–LDH/NiMo	1 M KOH	550	61	44
NiCoFe–LDH	1 M KOH	233	29.39	45
NiCo_2_O_4_	1 M KOH	340	75	46
NiFe–LDH/rGO	1 M KOH	245	—	47
Exfoliated CoCo–LDHs	1 M KOH	353	45	48
FeMoSe@NiCo–LDH	1 M KOH	240	78.4	49
CuCo_2_O_4_/NrGO	1 M KOH	360	64	50
Ni_1_Co_2_–LDH	1 M KOH	317	96.47	51
FeCo_2_S_4_@NiCo–LDH	1 M KOH	262	142.1	52
Co_9_S_8_/N,S-rGO	1 M KOH	266	75.5	53
Co_3_O_4_/Ppy/C	1 M KOH	340	87	54
NiMo–LDH@7%rGO	1 M KOH	230	60	This work

The kinetics of water oxidation can be evaluated using the Tafel slope, calculated by eqn (3): *η* = *a* + *b* log *j*. The Tafel slope determination is a commonly employed technique for finding the rate-determining step for OER. The Tafel slope values indicate the particular reaction route employed by the electrocatalytic process. The Tafel values for various catalysts are shown in Fig. 5(b). The modified GCE with NiMo–LDH@7%rGO demonstrates a minimum Tafel slope value of 60 mV dec^−1^, outperforming NiMo–LDH@3%rGO/GCE, NiMo–LDH@12%rGO/GCE and bare GCE, indicating that a smaller value of overpotential is required to sustain the reaction. Furthermore, a lower Tafel slope value indicates efficient OER at the surface as a consequence of fewer barriers to the progression of the reaction than the presence of numerous charged intermediates competing for the release of oxygen gas on the electrocatalyst surface that can lead to a higher Tafel slope.^34^ Similarly, Fig. 5(c) compares overpotential with reported electrocatalysts for OER. The Tafel slope analysis of NiMo–LDH@7%rGO/GCE indicated a slope close to the standard 40 mV dec^−1^, implying that this reaction is crucial in determining the overall rate of the reaction. This observation corresponds with the *η*, wherein a reduced Tafel slope value indicates a significant rise in *j* with a slight change in overpotential, as shown in Fig. 5(d).

**Fig. 6:**
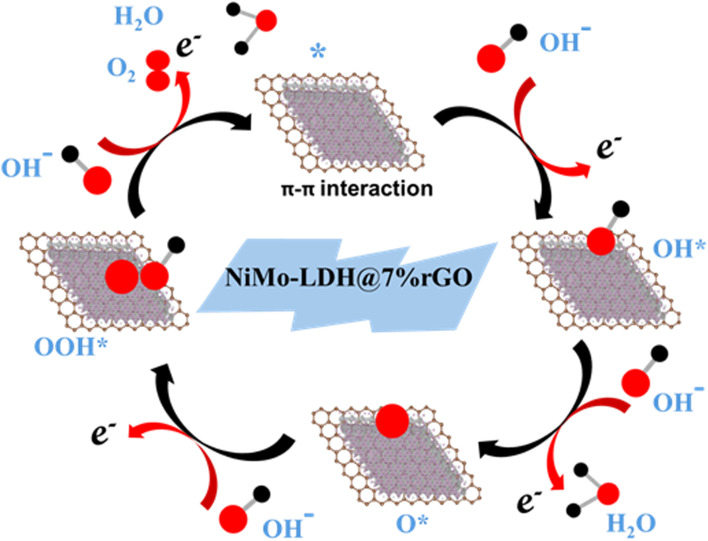
Proposed mechanism of the OER on the surface of NiMo–LDH@7% rGO.

Fig. 6 illustrates the overall proposed mechanism of the OER occurring on the surface of NiMo–LDH@7% rGO. In an alkaline environment, nickel sites undergo electrochemical oxidation to form Ni^3+^/Ni^4+^ species, which are recognized as active sites for the adsorption and oxidation of oxygen intermediates, thereby reducing the overpotential. For instance, research by Edvinsson *et al.* has shown that the redox activity of NiMoO_4_ nanorods on nickel foam is linked to γ-NiO(OH), which serves as the active surface species for efficient OER performance.^35^ Additionally, Huang *et al.* provided evidence through *operando* Raman spectroscopy and *ex situ* XRD that the unstable Ni(OH)_2_, when subjected to an applied potential, is reduced to form amorphous NiO, which subsequently converts into γ-NiO(OH).^36^ In a related study, Rajput *et al.* analyzed NiMoO_4_/NF in a 1 M KOH solution and identified a redox peak associated with the Ni(ii)/Ni(iii) transition. As cycling progressed, the current density increased, and the peak shifted anodically, indicating a reformation of the catalyst. After ten cycles, Raman spectra revealed bands at 483 and 562 cm^−1^ corresponding to NiO(OH), while immersion in KOH without an applied potential produced a 500 cm^−1^ Ni–O band. This suggests that both the alkaline environment and the applied potential facilitate the conversion of NiMoO_4_ to NiO/NiO(OH).^37^ Notably, some studies have identified NiMoO_4_ nanostructures on nickel foam (NF) as effective and stable catalysts for the OER.^38–40^ Zhang *et al.* demonstrated that within NiMoO_4_, molybdenum is oxidized to MoO_4_^2−^, which subsequently polymerizes and re-adsorbs onto the catalyst surface, thereby enhancing its stability. Furthermore, recent findings indicate that the Mo^6+^ ions located in the octahedral interstitial sites of the monoclinic NiMoO_4_ structure do not participate directly in the redox reactions. Instead, they play a crucial role in improving the material's electronic properties by enhancing conductivity, reducing electron density, promoting OH^−^ adsorption, and facilitating electron transfer, which supports effective Ni^2+^/Ni^3+^ redox reactions without faradaic contributions.^41,42^ In addition the incorporation of rGO not only improves conductivity and reduces charge-transfer resistance but also prevents the stacking of LDH nanosheets, resulting in an increased electrochemically active surface area. This effect was demonstrated by Chen *et al.*, who investigated the performance of NiFe–LDH@NF and NiFe–LDH/rGO@NF as catalysts for the OER. Their findings revealed that the rGO-enhanced material exhibited more pronounced absorption peaks for M–OH, M–O, and M–OOH intermediates compared to NiFe–LDH@NF. This indicates a higher concentration of intermediates and improved electron transfer, which collectively contribute to enhanced OER activity.^43^ Consequently, the overall mechanism for OER may need to be revised as follows,

10 NiMo–LDH@7%rGO + OH^−^ → NiMo@7%rGO–OH* + e^−^

11 NiMo–LDH@7%rGO–OH* + OH^−^ → NiMo@7%rGO–O* + H_2_O + e^−^

12 NiMo–LDH@7%rGO–O* + OH^−^ → NiMo@7%rGO–OOH* + e^−^

13 NiMo–LDH@7%rGO–OOH* + OH^−^ → O_2_ + H_2_O + e^−^

Similarly, Table 1 compares the current work with the previous reported catalysts, clearly showing that NiMo–LDH@7%rGO/GCE has the lowest Tafel slope and overpotential among the reported catalysts, exhibiting exceptional catalytic capabilities.

**Fig. 7:**
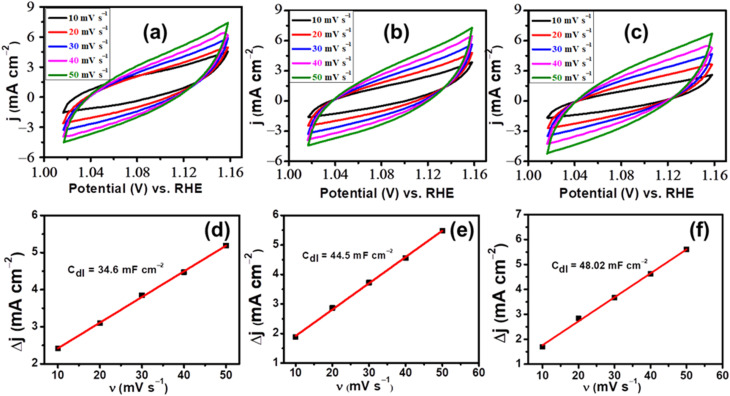
Cyclic voltammograms in non-faradic regions at (a) NiMo–LDH/GCE (b) NiMo–LDH@3%rGO/GCE (c) NiMo–LDH@7%rGO/GCE and double layer capacitances value (d) NiMo–LDH/GCE (e) NiMo–LDH@3%rGO/GCE (f) NiMo–LDH@7%rGO/GCE.

Besides overpotential and Tafel slope another crucial factor in assessing the efficiency of LDHs as electrocatalysts is their ECSA, defined as the accessible surface area for electrochemical processes. The porous nature of the LDHs nanomaterial determines the surface area for the adsorption of reactants and products, affecting the catalytic property of the LDHs. For example, an LDH with larger pores may exhibit a higher ECSA due to its capacity to absorb more reactants and products. The integration of rGO into the structure can influence the ECSA of the electrocatalysts since its high surface area and conductivity are shown to enhance the electron transport and augment the catalyst's overall efficiency. The ECSA of NiMo–LDH@7%rGO on a GCE in 1 M KOH solution at varying scan rates was investigated within a limited voltage range (1.00 V–1.17 V), depicted in Fig. 7(a)–(c). The relationship between scan rate and anodic–cathodic current difference (Δ*j*) in CV polarization curves, shown in Fig. 7(d)–(f), indicates that the *C*_dl_ value, which is half the slope, approximates the ECSA of NiMo–LDH and rGO supported NiMo–LDH. The *C*_dl_ of the NiMo–LDH@7%rGO is significantly greater than the NiMo–LDH and the NiMo–LDH@3%rGO. The ECSA of the NiMo–LDH@7%rGO exhibits a 1200.5 cm^2^ surface area, which is exceedingly greater than NiMo–LDH@3%rGO (1112.5 cm^2^) and NiMo–LDH (865 cm^2^). The results indicate that rGO promotes the transport of electrolytic ions to reactive sites, resulting in a reasonable ECSA value. An increase in its value indicates a higher number of reactive sites that facilitate the catalysis of water.

**Fig. 8:**
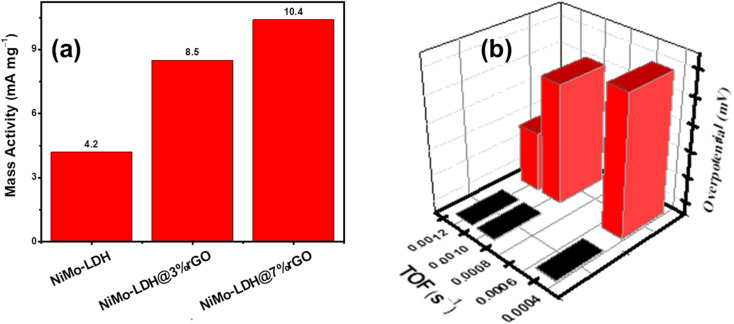
(a) Mass activity of synthesized catalysts: NiMO–LDH, NiMo–LDH@3%rGO, NiMo–LDH@7%rGO (b) relation of TOF with overpotentials.

To evaluate the intrinsic properties of synthesized electrocatalysts, mass activity (mA mg^−1^) was calculated at a fixed overpotential of 1.9 V by dividing the current density by mass loading. The values of MA of NiMo–LDH@7%rGO/GCE (10.4 mA mg^−1^) were higher than NiMo–LDH@3%rGO/GCE (8.5 mA mg^−1^) and NiMo–LDH/GCE (4.2 mA mg^−1^) as illustrated in Fig. 8(a). Additionally, the TOF was employed to determine the intrinsic activity of the catalyst as shown in Fig. 8(b), which was plotted against the applied voltage *vs.* RHE. The TOF value for electrocatalyst NiMo–LDH@7%rGO/GCE was calculated to be 0.0012 s^−1^, while the values for NiMo–LDH@3%rGO/GCE and NiMo–LDH/GCE were 0.0010 s^−1^, and 0.00049 s^−1^, respectively. This signifies that NiMo–LDH@7%rGO/GCE demonstrates a higher intrinsic activity dependent on surface reactions. These findings further highlight the fact that adding rGO enhanced the mass activity, specific activity, and turnover frequency. In addition, the exceptional efficiency of NiMo–LDH@7%rGO/GCE facilitates the OER because of the larger surface area of the nanomaterial, unobstructed channels, and porous layered structure. These variables enhance the transfer of ions to the active sites, leading to a significant enhancement in OER performance.

**Fig. 9:**
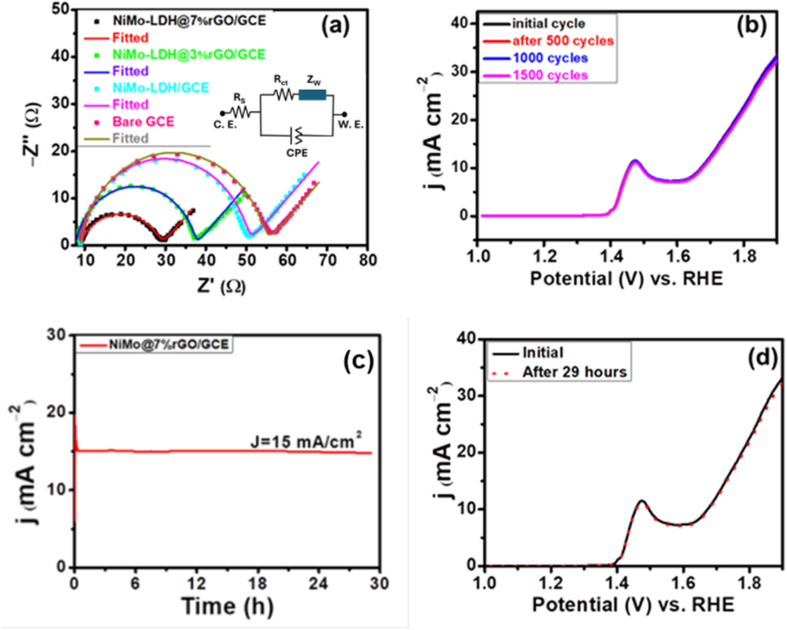
(a) Electrochemical impedance spectra at bare GCE, NiMo–LDH/GCE, NiMo–LDH@3%rGO/GCE, and NiMo–LDH@7%rGO/GCE, with the equivalent circuit model illustrated in the inset. (b) LSV plots before and after the 1500 cycles. (c) Chronoamperometry stability test for up to 29 hours. (d) LSV was obtained before and after 29 hours of chronoamperometric operation.

Furthermore, EIS was conducted to assess the electron transfer rate of each catalyst during the electrochemical reaction and to elucidate the processes behind OER activity for rGO supported bimetallic LDHs hybrid nanomaterial. The AC impedance of all materials was performed at 1.5 V in a voltage range of 0.1 Hz to 10^6^ Hz. Fig. 9(a) shows the fitted EIS spectra of the three as-prepared catalysts and bare GCE. The charge-transfer resistance *R*_ct_ of NiMo–LDH@7%rGO/GCE, NiMo–LDH@3%rGO/GCE, NiMo–LDH/GCE, and bare GCE was found to be 20.99 Ω, 29.47 Ω, 41.77 Ω, and 46.95 Ω, respectively. The results demonstrate that the NiMo–LDH@7%rGO/GCE exhibits much lower *R*_s_ of 8.95 Ω and *R*_ct_ of 20.99 Ω compared to other catalysts, signifying effective charge transfer between the electrode and catalyst. Fig. 9(a) depicts the intersection of Nyquist impedance curve with *Z*′ at high frequencies, pointing out *R*_s_ associated with the reaction rate, and at low frequencies, the *R*_ct_.^55^

Electrochemical stability is a pertinent parameter for evaluating catalyst performance, as operational conditions can affect the efficiency of the reaction that directly influences the stability of the catalyst. Chronoamperometry and CV experiments were conducted to assess the stability of the electrocatalyst. The stability of NiMo–LDH@7%rGO/GCE was assessed through a cycling endurance test, by subjecting the catalyst to repeated LSV cycles within the potential range of 0–1.5 V *vs.* RHE at a scan rate of 50 mV s^−1^. These curves were subsequently acquired on the modified electrode in a freshly prepared electrolyte. The LSV curves showed that current density decreased by only 3% of the original current density after 1500 cycles, as illustrated in Fig. 9(b). This indicated that the structure remained intact, and no variation in current density was observed. Further, chronoamperometry of NiMo–LDH@7%rGO/GCE was conducted in 1 M KOH solution at a corresponding potential of 1.46 V relative to Ag/AgCl. The 29 hour time frame indicates that the current density of 15 mA cm^−2^ remains constant up to a 29 hour period, which is illustrated in Fig. 9(c). The lack of change in current density shows the stability of the electrocatalyst during the operational conditions, offering sustained performance.

The stability evaluation was performed on NiMo–LDH@7%rGO/GCE by recording LSV before and after 29 hours of chronoamperometric operation. Fig. 9(d) shows notable stability, with a minor divergence in the LSV curve compared to the original curve after 29 hours of operation. This remarkable long-term stability can be attributed to the strong synergistic effect between Ni and Mo ions and the incorporation of rGO, which serves as an electronic scaffold to the electrocatalysts. These results indicate that NiMo–LDH@7%rGO/GCE displays exceptional catalytic performance, surpassing most TM-based nanomaterials employed for the OER in terms of overpotential, Tafel slope and current density.^56^

### 4.2 Water reduction reaction

The as-prepared bifunctional NiMo–LDH@rGO nanomaterial samples were further assessed for their electrocatalytic efficiency for HER. The rGO-functionalized LDHs have attracted much interest as prospective HER based catalysts owing to their porous architecture, which offer a substantial surface area for the reaction and allow for tunability. Also, rGO introduces pores and channels, which promotes the adsorption and desorption of hydrogen ions, hence improving mass transport and catalytic activity.

**Fig. 10:**
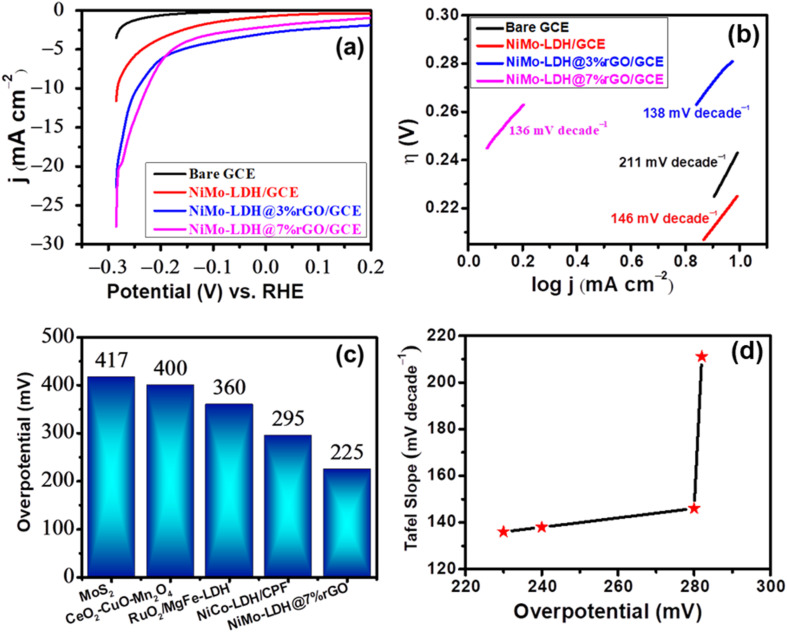
(a) LSV curves obtained at bare GCE, NiMo–LDH/GCE, NiMo@3% rGO/GCE, and NiMo–LDH@7% rGO/GCE in 0.5 M H_2_SO_4_. (b) Tafel slope analysis for the synthesized electrocatalysts in acidic medium. (c) Comparison of NiMo–LDH@7% rGO/GCE with reported HER electrocatalysts. (d) Relationship between Tafel slope and overpotential.

The electrocatalytic HER performance was evaluated for NiMo–LDH@rGO/GCE in 0.5 M H_2_SO_4_ solution for assessing its water-splitting efficiency as a catalyst. The LSV polarization curves are shown in Fig. 10(a). The NiMo–LDH@7%rGO/GCE has superior HER performance as compared to NiMo–LDH@3%rGO/GCE and NiMo–LDH/GCE. This can be ascribed to its high surface area and increased number of reactive sites, resulting in lower overpotential (225 mV) obtained for NiMo–LDH@7%rGO/GCE at 10 mA cm^−2^. Another parameter used to evaluate the efficiency of the HER was the current density. Here, maximum current density was achieved by NiMo–LDH@7%rGO/GCE due to enhanced exposure of active sites for electron transfer, accelerating reaction kinetics.^57^

The Tafel slope is illustrated in Fig. 10(b) which elucidates the mechanism of HER in acidic medium. The Tafel slope of NiMo–LDH@7%rGO/GCE is markedly lower at 136 mV dec^−1^, in comparison to NiMo–LDH/GCE (138 mV dec^−1^), NiMo–LDH@3%rGO/GCE (146 mV dec^−1^), and bare GCE (211 mV dec^−1^). This indicates that the HER for NiMo–LDH@7%rGO/GCE occurs through a Volmer–Heyrovsky mechanism with enhanced kinetics.^58^ In the Volmer phase, a proton from the electrolyte discharges towards the electrode by capturing electrons from the surface to form the H* adsorbed species (eqn (14)). During the Heyrovsky step, the adsorbed hydrogen atom is liberated from the surface and reacts with the proton (in the acidic medium) to generate a hydrogen gas molecule (eqn (15)). While in a separate step (Tafel step), two adsorbed H* species combine to form the H_2_ molecule (eqn (16)). Hence, the electrochemical conversion of the H_2_O molecule into H_2_ gas takes place through various distinct steps.^59^ The following mechanism is followed by HER in an acidic medium,

14 H^+^ + e^−^ → H*

15 H^+^ + H* + e^−^ → H_2_

16 2H* → H_2_

To gain deeper insights, the HER mechanism was investigated, which entails the reduction of protons through a series of steps that ultimately yield hydrogen gas.^60^ The HER mechanism can vary depending on whether the conditions are acidic or alkaline. In their study, Li *et al.* utilized voltage-dependent *operando* Raman spectroscopy to analyze the active site of N–NiMoO_4_/Ni/CNTs. Their findings revealed that the NiO peaks between 400 and 600 cm^−1^ remained unchanged with varying potential, indicating that NiO does not contribute to the reaction.^61^ In contrast, the spectral features related to Ni (1000–1200 cm^−1^) and C (1400–1600 cm^−1^) showed significant variations, with transient peaks for Ni^2+^–OH* and C–H* appearing at approximately 1150 and 1520 cm^−1^ at −0.1 V. This behavior suggests that water dissociation and its subsequent adsorption on Ni^2+^ are taking place.^62^ The disappearance of these peaks after the HER confirms their transient nature, while the MoO_4_^2−^ peaks at 814, 889, and 938 cm^−1^ remain stable, suggesting that MoO_4_^2−^ enhances conductivity without directly participating in the reaction. Conducting the HER study in an acidic environment provides sufficient H^+^ ions to adsorb onto the Ni^2+^ ions, facilitating their direct involvement in hydrogen evolution. Additionally, the rGO component functions as a co-catalyst, promoting hydrogen adsorption and stabilizing NiMo–LDH through effective electron transfer to active sites, thereby increasing the accessibility of the surface area.^63,64^Fig. 10(c) and (d) illustrate the comparison of reported electrocatalysts with the as-synthesized material and the relationship graph (between Tafel slopes and overpotentials of the as-prepared LDHs) respectively. Table 2 presents the performance of various electrocatalysts for the HER, highlighting the effectiveness of our synthesized rGO-based bimetallic NiMo–LDH.

**Table 2:** Comparison of the efficiency of as synthesized electrocatalysts with reported HER electrocatalyst

Catalysts	Electrolyte	Overpotential *η* (mV)	Tafel slope (mV dec^−1^)	Ref.
Co_3_O_4_/WO_3_/C nanorods	0.5 M H_2_SO_4_	400	105	65
NiCo–LDH/CFP	1 M KOH	295	195.51	66
NiFe–LDH/CMT	1 M KOH	333	140	67
RuO_2_/MgFe–LDH	0.5 M H_2_SO_4_	360	76.06	68
Fe–NiS_2_	0.5 M H_2_SO_4_	121	37	69
Ni–MoS_2_	0.5 M H_2_SO_4_	417	139	70
NiFePd–LDH	1 M KOH	412	171.4	71
C doped CoFe_2_O_4_/Fe_2_O_3_	1 M KOH	236	146	72
CeO_2_–CuO–Mn_3_O_4_	1 M KOH	380	175	73
Co_3_S_4_ doped MoS_2_–Ni_3_S_2_	1 M KOH	317	109	74
NiMo–LDH@7%rGO	0.5 M H_2_SO_4_	225	136	This work

**Fig. 11:**
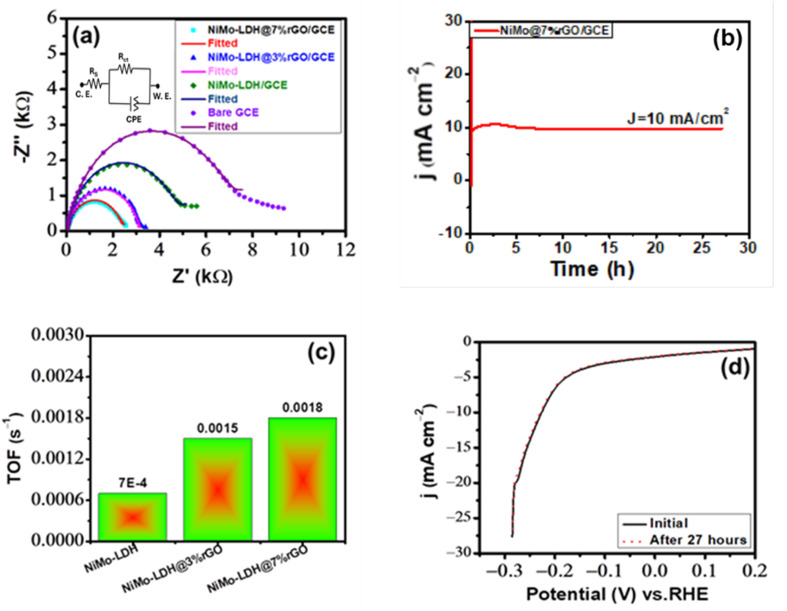
(a) Electrochemical impedance spectra recorded in 0.5 M H_2_SO_4_ solution, with corresponding Randel's circuit as shown in the inset. (b) Chronoamperometry test up to 27 hours. (c) TOF calculation for HER. (d) LSV performed before and after 27 hour chronoamperometric test.

Moreover, EIS measurements were conducted to examine the kinetics of the electrocatalysts at the electrode/electrolyte interface in 0.5 M H_2_SO_4,_ as illustrated in Fig. 11(a). All the fitted curves of EIS data were analyzed using a Randles equivalent circuit depicted in the inset of Fig. 11(a), which comprises of a *R*_s_ resistor and a series arrangement with resistor *R*_ct_ and constant phase element (CPE) that accounts for inhomogeneity at the electrode. According to the EIS results, the NiMo–LDH@7%rGO/GCE, NiMo–LDH@3%rGO/GCE, and NiMo–LDH/GCE exhibit an *R*_ct_ value of 2.54 kΩ, 2.84 kΩ, and 4.93 kΩ, respectively, while bare GCE has a value of 7.35 kΩ. From this EIS data, NiMo–LDH@7%rGO/GCE possesses the smallest *R*_ct_ = 2.54 kΩ, suggesting a fast charge transfer kinetics. This value is consistent with the low overpotential and Tafel slope for NiMo–LDH@7%rGO/GCE.^75^ Additionally, the intrinsic activity was evaluated by TOF for HER at an overpotential of 280 mV. A bar graph is presented in Fig. 11(c) to illustrate the practical application of NiMo–LDH/GCE, NiMo–LDH@3%rGO/GCE, and NiMo–LDH@7%rGO/GCE. The layered structure of NiMo–LDH@7%rGO/GCE demonstrated a significant TOF value of 0.0018 s^−1^, while the values for NiMo–LDH@3%rGO/GCE and NiMo–LDH/GCE were 0.0015 s^−1^ and 0.0007 s^−1^, respectively. The data indicates that NiMo–LDH@7%rGO/GCE exhibits significant intrinsic activity in a surface-dependent process. Similarly, long-term electrocatalytic stability is essential for the catalyst's suitability for diverse applications, including energy storage devices. Further chronoamperometry was performed to assess the long-term stability of the catalyst. Fig. 11(b) depicts chronoamperometric results of NiMo–LDH@7%rGO/GCE, conducted at a constant potential of −0.225 V relative to Ag/AgCl for 27 hours to test the stability of the electrocatalyst. The investigation indicates that the NiMo–LDH@7%rGO/GCE exhibits long-term stability at 10 mA cm^−2^ for up to 27 hours. Thus, the LSV curves for HER of NiMo–LDH@7%rGO/GCE illustrated in Fig. 11(d) demonstrate its stability with a negligible decrease in current density.

## 5. Conclusions

In this study, bifunctional hybrid nano-based electrocatalyst NiMo–LDH@rGO was synthesized using hydrothermal method. Structural analysis through FTIR and XRD confirmed its successful synthesis while FE-SEM analysis revealed the nanoporous morphology of the NiMo–LDH@rGO. The effect of graphene on the performance of HER and OER was investigated. The NiMo–LDH with 7% rGO content demonstrated promising water splitting activity, achieving low overpotentials of 230 mV for OER and 225 mV for HER at a current density of 10 mA cm^−2^ in acidic solution. Its Tafel slope (60 mV dec^−2^) for OER signifies a fast reaction kinetics. Furthermore, anchoring NiMo–LDH nanomaterial onto a conductive substrate such as rGO resulted in long-term stability of the electrocatalyst. This was confirmed by conducting chronoamperometry for 27 hours for HER and 29 hours for OER, along with an improvement in cyclic stability. ECSA showed a large electrochemically active surface area while the electrochemical impedance measures revealed a low *R*_ct_ value, resulting in effective interfacial charge transfer. The electric field developed at the heterojunction interface through the synergistic effect of the integrated components was critical for increasing the catalytic efficiency. These findings highlight the significant potential of these nanomaterials, particularly the NiMo–LDH@7%rGO heterostructure, for advanced electrochemical water-splitting applications.

## Conflicts of interest

The authors declare no conflict of interest regarding the publication of this manuscript.

## Supplementary Material

RA-015-D5RA04536C-s001

## Data Availability

All data generated or analysed are available in this published article and its SI file. Supplementary information contains the LSV polarization curve of NiMo-LDH@12% rGO. See DOI: https://doi.org/10.1039/d5ra04536c.

## References

[cit1] Song Y., Shahidehpour M., Rahman S., Brandon N., Strunz K., Lin J., Zhao Y. (2023). Utilization of energy storage and hydrogen in power and energy systems: viewpoints from five aspects. CSEE Journal of Power and Energy Systems.

[cit2] Lipman T. E. , Hydrogen production science and technology, Fuel Cells and Hydrogen Production: a Volume in the Encyclopedia of Sustainability Science and Technology, 2019, vol. 2, pp. 783–798

[cit3] Fraunhofer I. , Towards a GW Industry—Fraunhofer ISE Provides a Deep-in Cost Analysis for Water Electrolysis Systems, 2022

[cit4] Song J., Wei C., Huang Z.-F., Liu C., Zeng L., Wang X., Xu Z. J. (2020). A review on fundamentals for designing oxygen evolution electrocatalysts. Chem. Soc. Rev..

[cit5] Li C., Baek J.-B. (2019). Recent advances in noble metal (Pt, Ru, and Ir)-based electrocatalysts for efficient hydrogen evolution reaction. ACS Omega.

[cit6] Ying J., Chen J.-B., Xiao Y.-X., de Torresi S. I. C., Ozoemena K. I., Yang X.-Y. (2023). Recent advances in Ru-based electrocatalysts for oxygen evolution reaction. J. Mater. Chem. A.

[cit7] Aralekallu S., Lokesh K. S., Singh V. (2024). Advanced bifunctional catalysts for energy production by electrolysis of earth-abundant water. Fuel.

[cit8] Gaur A., Sharma J., Lim D. H., Lee H. I., Han H. (2025). Recent Advances in Electronic Structure Modifications of Layered Double Hydroxide (LDH) for the Water Splitting Application. ChemCatChem.

[cit9] Lu X., Xue H., Gong H., Bai M., Tang D., Ma R., Sasaki T. (2020). 2D layered double hydroxide nanosheets and their derivatives toward efficient oxygen evolution reaction. Nano-Micro Lett..

[cit10] Li Z., Lin G., Wang L., Lee H., Du J., Tang T., Ding G., Ren R., Li W., Cao X. (2024). Seed-assisted formation of NiFe anode catalysts for anion exchange membrane water electrolysis at industrial-scale current density. Nat. Catal..

[cit11] Altalhi A. A., Mohamed E. A., Negm N. A. (2024). Recent advances in layered double hydroxides (LDH)-based materials: Fabrications, modification strategies, characterization, promising environmental catalytic applications, and prospective aspects. Energy Adv..

[cit12] Ma W., Zhang Y., Wang B., Wang J., Dai Y., Hu L., Lv X., Dang J. (2024). Significantly enhanced OER and HER performance of NiCo-LDH and NiCoP under industrial water splitting conditions through Ru and Mn bimetallic co-doping strategy. Chem. Eng. J..

[cit13] Banhart F., Kotakoski J., Krasheninnikov A. V. (2011). Structural defects in graphene. ACS Nano.

[cit14] Zou X., Yakobson B. I. (2015). An Open Canvas 2D Materials with Defects, Disorder, and Functionality. Acc. Chem. Res..

[cit15] Wang K., Guo J., Zhang H. (2022). Synergistic effect of nanosheet-array-like NiFe-LDH and reduced graphene oxide modified Ni foam for greatly enhanced oxygen evolution reaction and hydrogen evolution reaction. Mater. Adv..

[cit16] Saha S. (2024). 2D stacking of graphene and boron nitride for efficient metal free overall water splitting. Int. J. Hydrogen Energy.

[cit17] Rashid J., Gilani K., Arif A., Saraj C. S., Li W., Xu M. (2024). Facile synthesis of NiCo@ rGO as bifunctional electrocatalyst for enhanced water splitting. Int. J. Hydrogen Energy.

[cit18] Janani G., Surendran S., Lee D. K., Shanmugapriya S., Lee H., Subramanian Y., Sim U. (2024). Aggregation induced edge sites actuation of 3D MoSe2/rGO electrocatalyst for high-performing water splitting system. Aggregate.

[cit19] Daniel S., Patil G., Budagumpi S., Laxmaiah B. B., Sannegowda L. K. (2025). Unleashing the Bifunctional Activity of Iron Phthalocyanine–Reduced Graphene Oxide Hybrid for Water Electrolysis. Energy Fuels.

[cit20] Giddaerappa, Kousar N., Deshpande U., Sannegowda L. K. (2024). Cobalt Phthalocyanine Based Metal–Organic Framework as an Efficient Bifunctional Electrocatalyst for Water Electrolysis. Energy Fuels.

[cit21] Peng W., Zhang W., Lu Y., Li W., He J., Zhou D., Hu W., Zhong X. (2024). Mo-doping and construction of the heterostructure between NiFe LDH and NiSx co-trigger the activity enhancement for overall water splitting. J. Colloid Interface Sci..

[cit22] Jin L., Wang Q., Wang K., Lu Y., Huang B., Xu H., Qian X., Yang L., He G., Chen H. (2022). Engineering NiMoO 4/NiFe LDH/rGO multicomponent nanosheets toward enhanced electrocatalytic oxygen evolution reaction. Dalton Trans..

[cit23] Hong C., Ji J., Huang J., Zhang Y., Li L. (2025). NiMo/NiFe-LDH heterostructured electrocatalyst for hydrogen production from water electrolysis. Mater. Lett..

[cit24] Boumeriame H., Da Silva E. S., Cherevan A. S., Chafik T., Faria J. L., Eder D. (2022). Layered double hydroxide (LDH)-based materials: A mini-review on strategies to improve the performance for photocatalytic water splitting. J. Energy Chem..

[cit25] Raveendran A., Chandran M., Dhanusuraman R. (2023). A comprehensive review on the electrochemical parameters and recent material development of electrochemical water splitting electrocatalysts. RSC Adv..

[cit26] Yang S., Zhang Z., Zhou J., Sui Z., Zhou X. (2019). Hierarchical NiCo LDH–rGO/Ni foam composite as electrode material for high-performance supercapacitors. Trans. Tianjin Univ..

[cit27] He Q., Jia W., Wu X., Liu J. (2025). Flexible hybrid capacitors based on NiMoS@ NiCo-LDH composites under variable work conditions. CrystEngComm.

[cit28] Luo D., Chen Y., Chen Y., Wei Z., Zhang L., Ye X., Wang Q., Ma L. (2023). Construction of NiCoS@ NiMo-LDH hierarchical composite for high-performance supercapacitors. Mater. Lett..

[cit29] Chen C., Zhang H., Yan R., Wu T., Sun S., Xu Y., Li H. (2025). Defect engineering induced nanostructure changes of NiMo-layered double hydroxides/MOF heterostructure on battery type charge storage. J. Power Sources.

[cit30] Pal D. B., Rathoure A. K., Singh A. (2021). Investigation of surface interaction in rGO-CdS photocatalyst for hydrogen production: an insight from XPS studies. Int. J. Hydrogen Energy.

[cit31] Kumar V., Swart H., Som S., Kumar V., Yousif A., Pandey A., Shaat S., Ntwaeaborwa O. (2014). The role of growth atmosphere on the structural and optical quality of defect free ZnO films for strong ultraviolet emission. Laser Phys..

[cit32] Jeghan S. M. N., Kim N., Lee G. (2021). Mo-incorporated three-dimensional hierarchical ternary nickel-cobalt-molybdenum layer double hydroxide for high-efficiency water splitting. Int. J. Hydrogen Energy.

[cit33] Wang B., Jiao S., Wang Z., Lu M., Chen D., Kang Y., Pang G., Feng S. (2020). Rational design of NiFe LDH@ Ni 3 N nano/microsheet arrays as a bifunctional electrocatalyst for overall water splitting. J. Mater. Chem. A.

[cit34] Zhai P., Xia M., Wu Y., Zhang G., Gao J., Zhang B., Cao S., Zhang Y., Li Z., Fan Z. (2021). Engineering single-atomic ruthenium catalytic sites on defective nickel-iron layered double hydroxide for overall water splitting. Nat. Commun..

[cit35] Dürr R. N., Maltoni P., Tian H., Jousselme B., Hammarstrom L., Edvinsson T. (2021). From NiMoO4 to γ-NiOOH: detecting the active catalyst phase by time resolved in situ and operando Raman spectroscopy. ACS Nano.

[cit36] Huang J., Li Y., Zhang Y., Rao G., Wu C., Hu Y., Wang X., Lu R., Li Y., Xiong J. (2019). Identification of key reversible intermediates in self-reconstructed nickel-based hybrid electrocatalysts for oxygen evolution. Angew. Chem..

[cit37] Rajput A., Adak M. K., Chakraborty B. (2022). Intrinsic lability of NiMoO4 to excel the oxygen evolution reaction. Inorg. Chem..

[cit38] Wang Z., Wang H., Ji S., Wang X., Zhou P., Huo S., Linkov V., Wang R. (2020). A high faraday efficiency NiMoO4 nanosheet array catalyst by adjusting the hydrophilicity for overall water splitting. Chem.–Eur. J..

[cit39] Zhang S., She G., Li S., Qu F., Mu L., Shi W. (2019). Enhancing the electrocatalytic activity of NiMoO4 through a post-phosphorization process for oxygen evolution reaction. Catal. Commun..

[cit40] Yin Z., Zhang S., Chen W., Xinzhi M., Zhou Y., Zhang Z., Wang X., Li J. (2020). Hybrid-atom-doped NiMoO 4 nanotubes for oxygen evolution reaction. New J. Chem..

[cit41] Dabir M., Masoudpanah S., Mamizadeh M. (2024). Ultrathin needle-like NiMoO4/MoO3 heterostructure for supercapacitor and overall water splitting applications. J. Energy Storage.

[cit42] Jabeen S., Kumar P., Samra K. S. (2024). Boosting the electrochemical characteristics of MnMoO4 nanoparticles for supercapacitor applications. J. Appl. Electrochem..

[cit43] Chen J., Liu C., Ren W., Sun J., Zhang Y., Zou L. (2022). Synergistic effect of NF and rGO in preparing 3D NiFe-LDH/rGO@ NF composites on electrocatalysts performance. J. Alloys Compd..

[cit44] Wu Y., Chen M., Sun H., Zhou T., Chen X., Na G., Qiu G., Li D., Yang N., Zheng H. (2025). Coupling Ir single atom with NiFe LDH/NiMo heterointerface toward efficient and durable water splitting at large current density. Appl. Catal., B.

[cit45] Su C., Wang D., Wang W., Mitsuzaki N., Shao R., Xu Q., Chen Z. (2024). Rational design of bimetallic metal-organic framework derived three-dimensional flower-like and porous NiCoFe LDH/NF electrocatalyst for electrochemical overall water splitting. J. Electroanal. Chem..

[cit46] Lv X., Zhu Y., Jiang H., Yang X., Liu Y., Su Y., Huang J., Yao Y., Li C. (2015). Hollow mesoporous NiCo 2 O 4 nanocages as efficient electrocatalysts for oxygen evolution reaction. Dalton Trans..

[cit47] Youn D. H., Park Y. B., Kim J. Y., Magesh G., Jang Y. J., Lee J. S. (2015). One-pot synthesis of NiFe layered double hydroxide/reduced graphene oxide composite as an efficient electrocatalyst for electrochemical and photoelectrochemical water oxidation. J. Power Sources.

[cit48] Song F., Hu X. (2014). Exfoliation of layered double hydroxides for enhanced oxygen evolution catalysis. Nat. Commun..

[cit49] Kalusulingam R., Mariyaselvakumar M., Mathi S., Arokiasamy S., Mikhailova T. S., Alexandrovich G. M., Pankov I. V., Jeffery A. A., Myasoedova T. N. (2024). Synergetic FeMoSe@ NiCo-LDH hybrid heterostructures as a stable and effective bifunctional catalyst for sustained overall water splitting and seawater splitting. J. Alloys Compd..

[cit50] Bikkarolla S. K., Papakonstantinou P. (2015). CuCo2O4 nanoparticles on nitrogenated graphene as highly efficient oxygen evolution catalyst. J. Power Sources.

[cit51] Cao H., Liu B., Bai J., Li C., Xu G. (2025). Interfacial engineering of hierarchical ultra-thin NiCo-LDH nanosheet superstructures nanofiber for water cracking electrocatalysis. J. Alloys Compd..

[cit52] Yang Z., Huang J., Li K., Wang L., She H., Wang Q. (2024). FeCo2S4@ NiCo-LDH heterostructures as self-supported electrode for highly efficient overall water splitting. J. Solid State Chem..

[cit53] Liu H., Xu C.-Y., Du Y., Ma F.-X., Li Y., Yu J., Zhen L. (2019). Ultrathin Co9S8 nanosheets vertically aligned on N, S/rGO for low voltage electrolytic water in alkaline media. Sci. Rep..

[cit54] Jayaseelan S. S., Bhuvanendran N., Xu Q., Su H. (2020). Co3O4 nanoparticles decorated Polypyrrole/carbon nanocomposite as efficient bi-functional electrocatalyst for electrochemical water splitting. Int. J. Hydrogen Energy.

[cit55] Karmakar A., Karthick K., Sankar S. S., Kumaravel S., Madhu R., Bera K., Dhandapani H. N., Nagappan S., Murugan P., Kundu S. (2022). Stabilization of ruthenium nanoparticles over NiV-LDH surface for enhanced electrochemical water splitting: an oxygen vacancy approach. J. Mater. Chem. A.

[cit56] Zheng K., Ren J., Li X., Li G., Jiao L., Xu C. (2022). Engineering crystalline CoMP-decorated (M= Mn, Fe, Ni, Cu, Zn) amorphous CoM LDH for high-rate alkaline water splitting. Chem. Eng. J..

[cit57] Chen J., Li Z., Li Z., Zhou Y., Lai Y. (2024). Lattice-matched spinel/layered double hydroxide 2D/2D heterojunction towards large-current-density overall water splitting. Appl. Catal., B.

[cit58] Mu Y., Ma R., Xue S., Shang H., Lu W., Jiao L. (2024). Recent advances and perspective on transition metal heterogeneous catalysts for efficient electrochemical water splitting. Carbon Neutralization.

[cit59] Singha Roy S., Madhu R., Bera K., Nagappan S., Dhandapani H. N., De A., Kundu S. (2024). Tuning the activity and stability of CoCr-LDH by forming a heterostructure on surface-oxidized nickel foam for enhanced water-splitting performance. ACS Appl. Mater. Interfaces.

[cit60] Dubouis N., Grimaud A. (2019). The hydrogen evolution reaction: from material to interfacial descriptors. Chem. Sci..

[cit61] Zhao L., Zhang Y., Zhao Z., Zhang Q.-H., Huang L.-B., Gu L., Lu G., Hu J.-S., Wan L.-J. (2020). Steering elementary steps towards efficient alkaline hydrogen evolution via size-dependent Ni/NiO nanoscale heterosurfaces. Natl. Sci. Rev..

[cit62] Huang C., Zhang B., Wu Y., Ruan Q., Liu L., Su J., Tang Y., Liu R., Chu P. K. (2021). Experimental and theoretical investigation of reconstruction and active phases on honeycombed Ni3N-Co3N/C in water splitting. Appl. Catal., B.

[cit63] Li G. L., Qiao X. Y., Miao Y. Y., Wang T. Y., Deng F. (2023). Synergistic effect of N-NiMoO4/Ni heterogeneous interface with oxygen vacancies in N-NiMoO4/Ni/CNTs for superior overall water splitting. Small.

[cit64] Wang H.-Y., Ren J.-T., Wang L., Sun M.-L., Yang H.-M., Lv X.-W., Yuan Z.-Y. (2022). Synergistically enhanced activity and stability of bifunctional nickel phosphide/sulfide heterointerface electrodes for direct alkaline seawater electrolysis. J. Energy Chem..

[cit65] Ahmed I., Dastider S. G., Biswas R., Roy A., Mondal K., Haldar K. K. (2024). Co3O4/WO3/C nanorods with porous structures as high-performance electrocatalysts for water splitting. ACS Appl. Nano Mater..

[cit66] Yang H., Zhou Z., Yu H., Wen H., Yang R., Peng S., Sun M., Yu L. (2023). Alkali treatment of layered double hydroxide nanosheets as highly efficient bifunctional electrocatalysts for overall water splitting. J. Colloid Interface Sci..

[cit67] Zou Y., Xiao B., Shi J.-W., Hao H., Ma D., Lv Y., Sun G., Li J., Cheng Y. (2020). 3D hierarchical heterostructure assembled by NiFe LDH/(NiFe) Sx on biomass-derived hollow carbon microtubes as bifunctional electrocatalysts for overall water splitting. Electrochim. Acta.

[cit68] Nagappan S., Jayan R., Rajagopal N., Krishnan A. V., Islam M. M., Kundu S. (2024). Tailoring Mott− Schottky RuO2/MgFe-LDH Heterojunctions in Electrospun Microfibers: A Bifunctional Electrocatalyst for Water Electrolysis. Small.

[cit69] Yan J., Wu H., Chen H., Jiang R., Liu S. F. (2017). Fe (III) doped NiS 2 nanosheet: a highly efficient and low-cost hydrogen evolution catalyst. J. Mater. Chem. A.

[cit70] Kuang P., Tong T., Fan K., Yu J. (2017). In situ fabrication of Ni–Mo bimetal sulfide hybrid as an efficient electrocatalyst for hydrogen evolution over a wide pH range. ACS Catal..

[cit71] Liu D., Liu J., Xue B., Zhang J., Xu Z., Wang L., Gao X., Luo F., Li F. (2023). Bifunctional water splitting performance of NiFe LDH improved by Pd2+ doping. ChemElectroChem.

[cit72] Farooq A., Khalil S., Basha B., Habib A., Al-Buriahi M., Warsi M. F., Yousaf S., Shahid M. (2024). Electrochemical investigation of C-doped CoFe2O4/Fe2O3 nanostructures for efficient electrochemical water splitting. Int. J. Hydrogen Energy.

[cit73] Jafari S., Shaghaghi Z. (2025). Engineering active sites in ternary CeO2-CuO-Mn3O4 heterointerface embedded in reduced graphene oxide for boosting water splitting activity. Sci. Rep..

[cit74] Muthurasu A., Ojha G. P., Lee M., Kim H. Y. (2020). Zeolitic imidazolate framework derived Co3S4 hybridized MoS2–Ni3S2 heterointerface for electrochemical overall water splitting reactions. Electrochim. Acta.

[cit75] Jiang H., Yu Y., Duan X., Chen P., Wang S., Qiu X., Ye L., Tu X. (2024). Heterostructured MoO3 anchored defect-rich NiFe-LDH/NF as a robust self-supporting electrocatalyst for overall water splitting. Small.

