# Supplementary material for: Bimetallic layered-double hydroxides anchored on reduced graphene oxide as a bifunctional electrocatalyst for electrochemical water splitting
Source: RSC Adv. 2025 Sep 18;15(41):34105–21. doi: 10.1039/d5ra04536c (PMC12444307; doi:10.1039/d5ra04536c)
Supplement: RA-015-D5RA04536C-s001 [file RA-015-D5RA04536C-s001.pdf]

Supplementary Information (SI) for RSC Advances.
This journal is © The Royal Society of Chemistry 2025

Supporting Information

Bimetallic Layered-Double Hydroxides Anchored on Reduced Graphene Oxide as a Bifunctional Electrocatalyst for Electrochemical Water Splitting

Asad Ullah Khan[1], Syed Haider Ali Shah[1], Fariah Salam[2], Afzal Shah*[1], Faiza Jan Iftikhar[3], Muhammad Umar Farooq[4], Muhammad Abdullah Khan[2]

[1]Department of Chemistry, Quaid-i-Azam University, Islamabad 45320, Pakistan
[2]Renewable Energy Advancement Laboratory, Department of Environmental Sciences, Quaid-i-Azam University, Islamabad 45320, Pakistan

[3]NUTECH School of Applied Science & Humanities, National University of Technology, Islamabad 44000, Pakistan
[4]National Center for Physics, Islamabad, 45320, Pakistan

Correspondence*: afzals_qau@yahoo.com

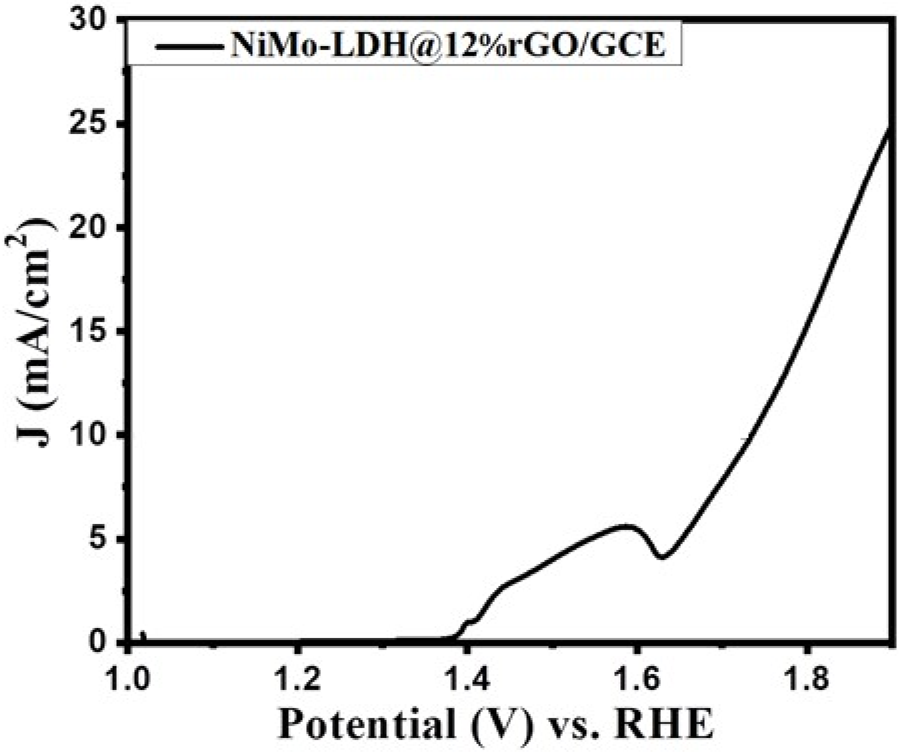

Figure S1. LSV polarization curve of NiMo-LDH@12%rGO
